# Adaptive Differentiation in Seedling Traits in a Hybrid Pine Species Complex, *Pinus densata* and Its Parental Species, on the Tibetan Plateau

**DOI:** 10.1371/journal.pone.0118501

**Published:** 2015-03-10

**Authors:** Jingxiang Meng, Jian-Feng Mao, Wei Zhao, Fangqian Xing, Xinyu Chen, Hao Liu, Zhen Xing, Xiao-Ru Wang, Yue Li

**Affiliations:** 1 State Engineering Laboratory of Forest Tree Breeding, Key Laboratory of Genetic and Breeding in Forest Trees and Ornamental Plants, Beijing Forestry University, Beijing, China; 2 College of Biology Sciences and Technology, Beijing Forestry University, Beijing, China; 3 State Key Laboratory of Systematic and Evolutionary Botany, Institute of Botany, Chinese Academy of Sciences, Beijing, China; 4 College of Resources and Environment, College of Agriculture and Animal Husbandry of Tibet University, Linzhi, Tibet, China; 5 Department of Ecology and Environmental Science, Umeå University, Umeå, Sweden; USDA Forest Service, UNITED STATES

## Abstract

Evidence from molecular genetics demonstrates that *Pinus densata* is a natural homoploid hybrid originating from the parent species *Pinus tabuliformis* and *Pinus yunnanensis*, and ecological selection may have played a role in the speciation of *P*. *densata*. However, data on differentiation in adaptive traits in the species complex are scarce. In this study, we performed a common garden test on 16 seedling traits to examine the differences between *P*. *densata* and its parental species in a high altitude environment. We found that among the 16 analyzed traits, 15 were significantly different among the species. *Pinus tabuliformis* had much earlier bud set and a relatively higher bud set ratio but poorer seedling growth, and *P*. *yunnanensis* had opposite responses for the same traits. *P*. *densata* had the greatest fitness with higher viability and growth rates than the parents. The relatively high genetic contribution of seedling traits among populations suggested that within each species the evolutionary background is complex. The correlations between the seedling traits of a population within a species and the environmental factors indicated different impacts of the environment on species evolution. The winter temperature is among the most important climate factors that affected the fitness of the three pine species. Our investigation provides empirical evidence on adaptive differentiation among this pine species complex at seedling stages.

## Introduction

Natural hybridization plays an important role in plant evolution [1–3]. Hybridization facilitates adaptive evolution and generates new species either through allopolyploid or homoploid hybrid speciation (HHS) [4–6]. HHS is hybridization that occurs in the wild without a change in the chromosome number [7,8]. According to previous studies, HHS is accomplished by the combination of fertility success and ecological selection following initial hybridization [9–11]. Homoploid hybrids are usually found in novel habitats that are not occupied by parental species, where they are generally believed to have higher fitness than the parent species [12–14]. To assess whether differential adaptation to an environment has played a role during species diversification, field experiments with natural selection on fitness-related traits could be helpful in understanding how hybrid species occupy the hybrid zone and how populations within the species maintain coherence or divergence from the initial homoploid hybridization.

*Pinus densata* represents one of the most documented cases of HHS, with important evolutionary consequences [15–17]. This species forms extensive pure forests that regenerate on the southeastern Tibetan Plateau at elevations ranging from 2700 to 4200 m above sea level [9,17]. Molecular genetic evidences have indicated that *P*. *densata* originated from hybridization between *Pinus tabuliformis* and *Pinus yunnanensis* (Wang 2011; Gao 2012). *Pinus tabuliformis* is widely distributed in northern and central China with an area range spanning the temperate arid steppe zone, while *P*. *yunnanensis* is relatively limited to southwest China in isolated subtropical monsoon broadleaf evergreen forests [8,18–21]. These three pine species are distributed geographically by latitude with *P*. *tabuliformis*, *P*. *densata*, and *P*. *yunnanensis* generally found in northern, intermediate, and southern regions, respectively [9]. The northeastern part of the *P*. *densata* range overlaps with the range of *P*. *tabuliformis*, and the central region of *P*. *densata* overlaps with the range of *P*. *yunnanensis* [17]. The evolution of *P*. *densata*, following the initial hybridization event into a stable taxonomic unit, was promoted by uplift of the Tibetan Plateau, after which the successful hybrid colonized the new, empty plateau habitat that was originally inaccessible to both parent species [16,22,23]. Previous studies based on maternally inherited mitochondrial (mt) and paternally inherited chloroplast (cp) DNA sequence variation, revealed that the extensive distribution of *P*. *densata* on the plateau was caused by multiple waves of colonization from an ancient hybrid zone located in the northeast periphery of its current distribution [12]. These waves of westward colonization created three clusters of populations along the route of colonization: the east group, the central group, and the west group. Additionally, the direction and intensity of introgressions from the two parent species varied among geographical regions [10,12].

*Pinus densata* has colonized a vast area on the Tibetan Plateau [9,10,12]. Previous authors hypothesized that ecological selection may have promoted the evolution of *P*. *densata* [10,12]. However, no field evidence exists to support this theory of speciation. To explore the fitness variation of *P*. *densata* compared with its parent species at higher elevations, we conducted a transplantation experiment on the Tibetan Plateau with seedlings from *P*. *densata*, *P*. *tabuliformis*, and *P*. *yunnanensis*. The objectives of this study were (1) to examine differences in fitness at seedling stages between the hybrid species and its parent species on the Tibetan Plateau, and (2) to examine the fitness variation among parental populations, with the possibility that some parental populations may be better adapted in the hybrid habitat, These investigations will increase our knowledge of conifer species divergence through adaptive evolution.

## Materials and Methods

### Study system

**Fig 1:**
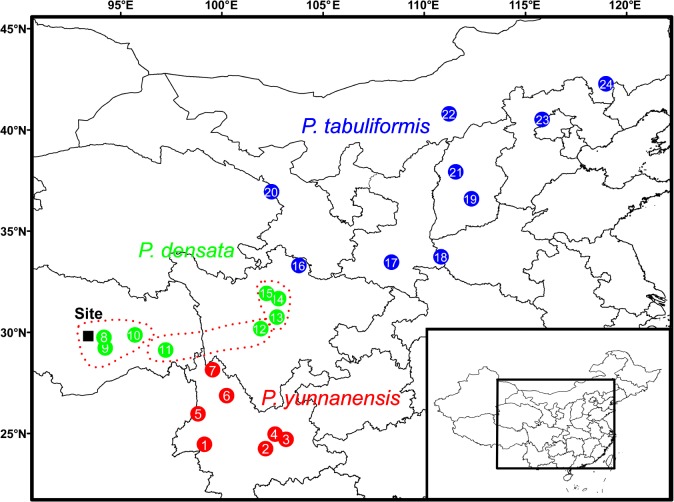
Geographic distribution of the 24 populations included in the field experiment. The three P. densata groups identified by previous genetic analyses are described as Group East, Group Central and Group West.

Field experiment was conducted in Linzhi, Tibet (29iment was conduc2900 m in altitude), which represents a typical habitat for *P*. *densata* (Fig. 1). The area is in the plateau semi-humid monsoon climate zone with freezing winters and seasonal drought [24,25]. Meteorological data including the temperature, precipitation, air pressure, and sunshine duration during the experiment (April 2011–March 2013) were collected from the China Meteorological Administration (Table 1).

**Table 1:** Meteorological data of the field experiment in Linzhi, Tibetan, during the field test.

Year	Average temperature (°C)	Maximum temperature (°C)	Minimum temperature (°C)	July temperature (°C)	January temperature (°C)	Average Surface Temperature (°C)	Precipitation (mm)	Barometric pressure (hp)	Sunshine duration (h)
1st	9.6	29.1	−10.2	17.5	4.4	13.3	735.5	709	1998.1
2nd	9.4	28.9	−13.6	16.4	3.1	13.6	530.4	709.8	1998.5

The meteorological data of each year were collected from China Meteorological Data Sharing Service System (http://www.cma.gov.cn/2011qxfw/2011qsjgx/)

1^st^ year from April 2010 to March 2011, 2^nd^year from April.2011 to March 2012.

Twenty-four natural populations, 9 of *P*. *tabuliformis*, 8 of *P*. *densata*, and 7 of *P*. *yunnanensis*, were used to represent populations found in the primary geographical regions of each species (Table 2 and Fig. 1). Seeds were collected from each population when cones ripened naturally. In each sample population, 30 or more individuals (separated by at least 50 m) were selected for cone collection. More than 3000 seeds for each population were mixed in separate cloth bags and stored under low temperature and humidity conditions in darkness before sowing. Only full and healthy seeds (determined by X-ray analyses) were used in the plantation experiment.

**Table 2:** Geographic location and the meteorological data of the sampling populations of *P*. *densata*, *P*. *tabuliformis* and *P*. *yunnanensis*.

Species		Populations	Longitude (N)	Latitude (E)	Elevation (m)	AT (°C)	AP (mm)	ATJ (°C)
*P*. *yunnanensis*								
	1	Baoshan, Dianxi	99°28′	24°28′	1897	14.3	1223	7.7
	2	Yuxi,Dianzhong	102°15′	24°15′	1849	17.5	995	10.9
	3	Yiliang, Dianzhong	103°43′	24°43′	1846	16	1007	9
	4	Kunming, Dianzhong	102°58′	24°58′	2242	14.1	1028	7.5
	5	Gongshan,Dianxi	98°58′	25°58′	1616	16.6	1436	9.5
	6	Lijiang, Dianxi	100°53′	26°53′	2493	13.3	970	6.5
	7	Zhangdian, Yunnan	99°01′	28°09′	3048	5.7	723	−1.8
*P*. *densata*								
Group West	8	Niyanghe, Tibetan	94°44′	29°45′	3203	8.5	605	−0.3
	9	Milin, Tibetan	94°14′	29°14′	2960	10	743	1.6
	10	Palongzangbu, Tibetan	95°55′	29°52′	2804	9.3	831	0.6
Group Central	11	Chayu, Tibetan	97°80′	29°08′	3264	7.4	797	−0.8
	12	Kangding, Sichuan	101°11′,	30°11′	2944	6.5	828	−2.5
Group East	13	Baoxing, Sichuan	102°49′	30°45′	2330	8.6	867	−0.8
	14	Lixian, Sichuan	102°40′	31°40′	2765	7.5	841	−1.7
	15	Maerkang, Sichuan	102°55′,	31°55′	2709	8	754	−1.2
*P*. *tabuliformis*								
	16	Jiuzhaigou, Sichuan	103°18′	33°18′	2393	6.7	658	−2.5
	17	Ningshan, Shannxi	108°29′	33°28′	1423	9.3	918	−2.5
	18	Lushi, Henan	110°44′	33°44′	1713	7.3	928	−4.8
	19	Lingkongshan, Shanxi	112°37′	36°36′	1664	8.4	571	−6.6
	20	Huzhu, Qinghai	102°58′	36°57′	2299	2.3	395	−9.5
	21	Fangshan, Shanxi	111°56′	37°56′	1941	4.3	528	−11.7
	22	Tumed, Inner Mongolia	111°47′	40°48′	1219	4.2	358	−14.6
	23	Songshan, Beijing	115°31′	40°31′	885	6.4	456	−10.7
	24	Ningcheng, Neimeng	118°17′	42°17′	1300	7.6	360	−10.4

The meteorological data of each selected populations were collected from WorldClim [62].

## Experimental design

The experimental site was located in the nursery at Tibet University. Plants were cultivated in seedling beds with forest soil, and seedlings from the different populations were distributed according to a randomized complete block design. 50 seeds from each population were sown in a population plot with the spacing of 10cm×10 cm. Four replication blocks, each with the full set of populations, were exposed to natural selection factors without human intervention (except weeding in the autumn).

We examined 16 quantitative traits involving growth, survival, phenology and needle color variation (Table 3). Surviving plants were measured from the approximate time of bud sprouts (April) to the time of bud set (October) over two years from early April 2011 to late March 2013. The stem height (SH, cm) and stem diameter 2 cm above the ground (D0, mm) were measured on 10 surviving individuals randomly from each plot (all plants in a plot were measured if the number of surviving plants was less than 10). Seedling phenology and viability traits that were measured for each experimental plot included the germination rate (GR), the survival rate (SR) in October, the bud set rate (BSR) in October, the survival rate after winter (SRAW) in April, and the second year-growth rate (SGR) in October. The ratios of seedlings with different needle colors (RLC) were determined after frost in October; the seedling colors observed were green, yellow, purple, and red.

**Table 3:** 16 fitness-related characteristics involving growth, viability, phenology and needle color.

Abbreviation	Traits	Correspond to fitness
Germinate		
GR	germination rate	High value with good reproduction
viability		
SR of the 1st year (%)	survival rate in autumn of 2011	High value with good viability
SRAW of the 1st year (%)	survival rate after winter of 2011	High value with good viability
SR of the 2nd year (%)	survival rate in autumn of 2012	High value with good viability
SRAW of the 2nd year (%)	survival rate after winter of 2012	High value with good viability
Phenology		
BSR of the 1st year (%)	bud set rate in october of 2011	High value with early bud set
BSR of the 2nd year (%)	bud set rate in october of 2012	High value with early bud set
SGR in the 2nd year (%)	second-growth rate in October of 2012	High value with long growth circle
Needle colors		
RLC in red (%)	ratios of seedlings with needle of red colors	High value with good viability and physiology changed
RLC in yellow (%)	ratios of seedlings with needle of yellow colors	High value with poor viability
RLC in purpul (%)	ratios of seedlings with needle of purpul colors	High value with good viability and physiology changed
RLC in green (%)	ratios of seedlings with needle of green colors	High value with good viability
Growth rate		
SH of the 1st year（cm）	stem height in october of 2011	High value with fast growth
SH of the 2nd year (cm)	stem height in october of 2012	High value with fast growth
D0 of the 1st year (mm)	stem diameter in october of 2011	High value with fast growth
D0 of the 2nd year (mm)	stem diameter in october of 2012	High value with fast growth

### Statistical analyses

To examine the effects of species and populations for each trait, we used a two-way nested analysis of variance (ANOVA) followed by Duncan’s multiple range test with block and species as fixed factors and population as a random factor. Variables were logit-transformed to ensure data had normal distributions prior to ANOVA and Duncan’s analyses. The general statistics for each trait in the population included the mean and the maximum and minimum values, which were used to describe the differences among species. The descriptions of each trait within each population were made using medians, quartiles, and amplitudes based on the average of each plot. All analyses were performed in the R (version 2.13.0) statistical and programming environment using the vegan package [26].

To evaluate the underlying dimensionality of the variables and to obtain an overview of the dominant patterns, we used PCA with a standardized matrix containing data on measured traits. Growth indicators were not considered in PCA for unavailable data from dead plants. Analysis using the ade4 package was performed using R statistics [27]. The PCA scores of the 1^st^ four components were used to calculate the species distance and a scatter diagram was generated using R-2.13.0 (ggplot2) to describe the dimensionality of the three pines in the 1^st^ two components.

Cluster analysis using arithmetic averages was performed for the 24 sample populations of the three pine species to estimate the similarity of populations for fitness related traits. Following correlation analyses among all indicators, 10 unrelated variables (SH of the 1st year, D0 of the 2nd year, GR and SR of the 1st year, SRAW of the 1st year, BSR of the 2nd year, RLC of the green, RLC of yellow, RLC of purple, and RLC of red) with correlation coefficients lower than 0.6 were chosen for cluster analysis. Cluster analysis was performed using the ward method in R statistics, and the Euclidean distances among populations were calculated from z-scores.

To examine habitat effects and adaptability variation, we calculated Pearson correlations between measured variables and primary habitat factors for the 24 sample populations of *P*. *densata*, *P*. *tabuliformis*, and *P*. *yunnanensis*. Data on three meteorological factors covering the last 50 years were collected (WorldClim, www.worldclimate.com), including three geographic factors (latitude, longitude, and elevation) and three meteorological factors (annual average temperature, annual precipitation, and average temperature in January). This multivariate correlation analysis was performed using SPSS 18.0 [28].

## Results

### Differentiation among the three pine species in the *P*. *densata* habitat on the Tibetan Plateau

Because of the high elevation of the Tibetan Plateau, 15 traits differed significantly among *P*. *densata*, *P*. *tabuliformis*, and *P*. *yunnanensis* (Table 4). *P*. *densata* possessed higher values of SRAW, SR, SGR, RLC in purple and SH, but displayed intermediate values between parent species for BSR, D0, GR, RCL in yellow and red. *P*. *yunnanensis* had the lowest values for traits of GR, SR and SRAW but had the highest value of D0. *P*. *tabuliformis* possessed intermediate. values of GR, SR in the 1st year, RLC in green of the three pine apecies to conditions on the Tibetan Plateau. BSR over 2 years for *P*. *tabuliformis* were higher than in the two other pine species, while RLC in red, D0, SH and the SGR in the 2nd year were lower than in the other species.

**Table 4:** Descriptive statistics and ANOVA of measured traits.

Species	Mean (Min∼Max)			Variance component(%)			
Statistics	*P*. *tabuliformis*	*P*. *densata*	*P*. *yunnanensis*	Block(d.f.)	Species(d.f.)	Pop/species(d.f.)	Residuals(d.f.)
GR	0.82(0.72 ∼0.96)^a^	0.69(0.49 ∼0.83)^b^	0.63(0.39 ∼0.78)^b^	0.65(3)	30.89(2)**	44.86(21)**	23.59(69)
SR of the 1^st^ year (%)	0.41(0.23 ∼0.6)^a^	0.32(0.14 ∼0.53)^b^	0.25(0.15 ∼0.32)^b^	3.57(3)	31.13(2)**	27.58(21)*	37.72(69)
SRAW of the 1^st^ year (%)	0.11(0.05 ∼0.17)^a^	0.15(0.03 ∼0.31)^a^	0.10(0 ∼0.17)^a^	2.21(3)	19.46(2)**	24.21(21)	53.64(69)
SR of the 2^nd^ year (%)	0.10(0.05 ∼0.14)^a^	0.14(0.03 ∼0.27)^a^	0.09(0 ∼0.17)^a^	1.47(3)	18.95(2)**	23.89(21)	55.69(69)
SRAW of the 2^st^ year (%)	0.10(0.05 ∼0.14)^a^	0.12(0.03 ∼0.24)^a^	0.08(0 ∼0.14)^a^	1.92(3)	6.54(2)*	46.56(21)*	44.99(69)
BSR of the 1^st^ year (%)	0.86(0.43 ∼1.00)^a^	0.29(0 ∼1)^b^	0.04(0 ∼0.25)^c^	0.59(3)	49.75(2)**	41.91(21)**	7.76(69)
BSR of the 2^nd^ year (%)	0.89(0.58∼1)^a^	0.52(0.23∼1)^b^	0.23 (0.04∼0.74)^c^	1.70(3)	13.08(2)**	33.52(20)	51.71(55)
SGR in the 2^nd^ year (%)	0.03(0∼ 0.10)^b^	0.15(0∼ 0.32)^a^	0.10(0∼0.24)^ab^	0.93(3)	10.26(2)**	28.86(21)*	59.95(69)
RLC in red (%)	0.01(0∼0.09)^c^	0.17(0∼0.46)^b^	0.23(0.09∼0.52)^a^	2.39(3)	21.97(2)**	36.65(21)**	38.98(69)
RLC in yellow (%)	0.38(0.19∼0.60)^a^	0.19(0∼0.53)^b^	0.01(0∼0.08)^c^	2.81(3)	38.52(2)**	31.03(21)*	27.64(69)
RLC in purpul (%)	0.15(0.04∼0.26)^ab^	0.20(0.05∼0.36)^a^	0.11(0.04∼0.23)^b^	1.66(3)	7.01(2)*	31.66(21)	59.68(69)
RLC in green (%)	0.46(0.29∼0.61)^b^	0.43(0.20∼0.73)^b^	0.64(0.32∼0.83)^a^	1.56(3)	13.88(2)**	50.47(21)**	34.09(69)
SH of the 1^st^ year（cm）	1.41(0.93∼2.05)^b^	1.98(1.30∼2.79)^b^	1.75(1.20∼2.22)^a^	6.24(3)	11.97(2)**	32.76(20)*	49.04(55)
SH of the 2^nd^ year (cm)	7.45(5.70∼10.16)^b^	9.26(5.77∼12.49)^ab^	7.97(6.63∼10.07)^a^	8.21(3)*	8.05(2)**	35.23(20)*	48.51(55)
D0 of the 1^st^ year (mm)	1.79(1.64∼1.96)^b^	1.79(1.47∼2.08)^a^	2.25(1.75∼2.72)^a^	5.37(3)	24.34(2)**	27.64(20)	42.64(55)
D0 of the 2^nd^ year (mm)	4.8(4.3∼5.7)^b^	5.2(3.9∼6.2)^a^	5.5(4.1∼7.2)^ab^	6.20(3)	6.74(2)	28.98(20)	58.09(55)

Mean with different superscript letters (a, b, c) are significantly different (*P* < 0.05) as determined by Duncan multiple range tests.

Data in the table: mean ± sd.

* *P* < 0.05;

** *P* < 0.01.

d.f., degrees of freedom.

**Fig 2:**
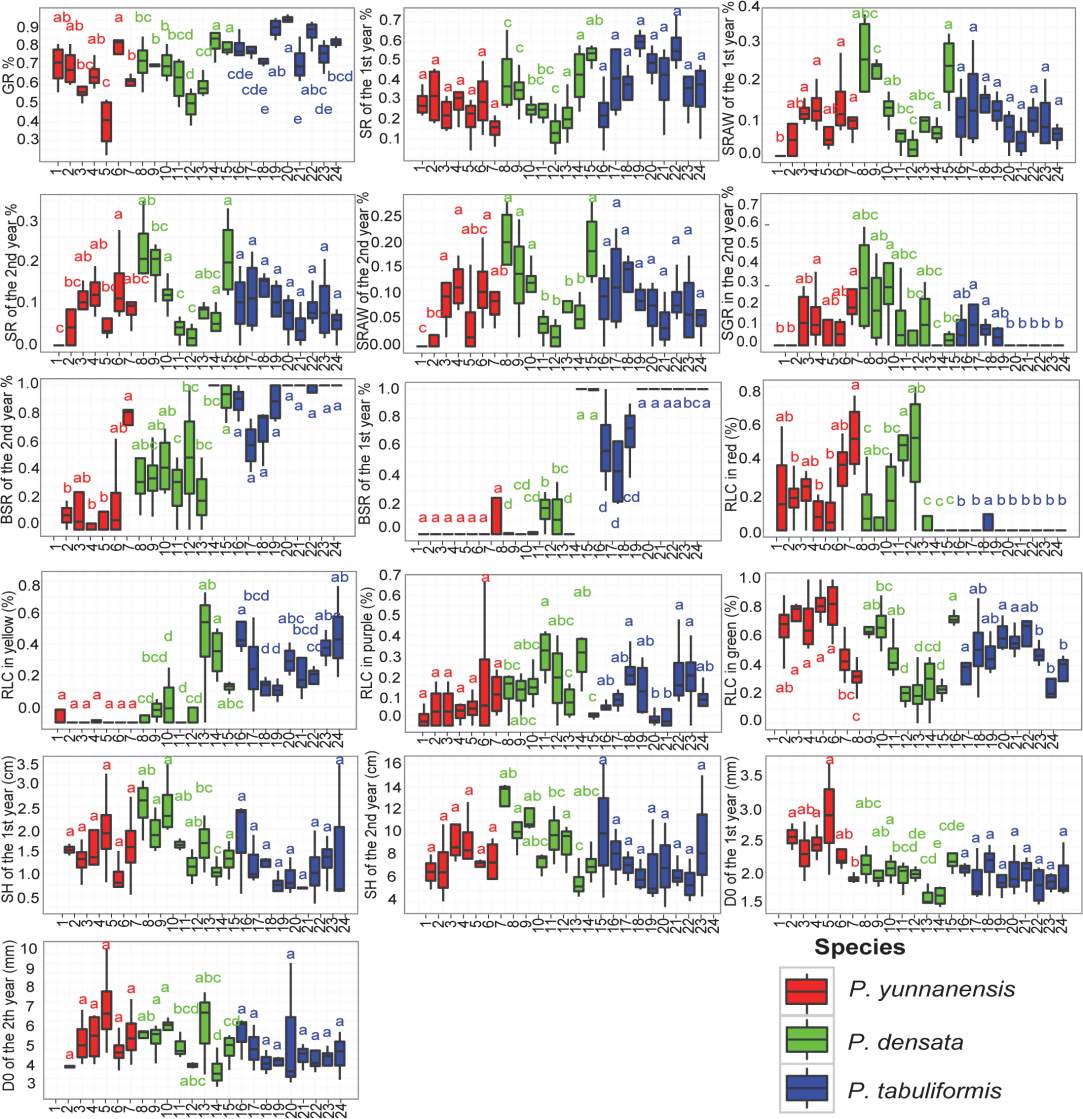
Box-plots of the 16 indices between populations and among species in the seedling stage. The rectangle region is the main part of the box-plot. The three upper, middle and lower lines represent the 75th, 50th and 25th percentile value of the variables, respectively. The vertical line in the middle of the box-plot is the tentacles line. The upper and lower ends of the line represent the maximum and minimum values of the variable, respectively, except for single or extreme values. “.”represents the single value, where the variable value exceeds 1.5 times the difference between the 75th percentile and 25th percentile. “*” represents the extreme value, where the variable value exceeds 3 times the difference between the 75th percentile and 25th percentile. Boxplots with different superscript letters (a, b, c) are significantly different (P < 0.05) as determined by Duncan multiple range tests.

Significant differences among populations were observed for *P*. *densata* (Table 4 and Fig. 2). This hybrid species had broader variations in SR in the 1^st^ year, SRAW in the 1^st^ year, BSR in the 1^st^ year, SGR in the 2^nd^ year, SH over 2 years, and RCL in four colors than parent species. Distributed in the northeast, population 15 had the highest GR, SR in the 1st year, and BSR in the 2nd year. Central populations 11 and 12 had the lowest survival rate but had high RCL in red seedlings. And the western populations 8, 9 and 10, had the best survival ratios and the fastest growth. Remarkably, population 15 had excellent seedling survivability, which was similar to the western populations.

For the two parental species, *s*ignificant differences were observed among geographic populations within a species for 10 traits (Table 4 and Fig. 2). *P*. *tabuliformis* had less variation for each trait than *P*. *densata*. In population 24, BSR of the 1^st^ year was the highest. Population 18 had higher fitness and a higher SR in the 2^nd^ year. Populations 16 and 17 had the lowest BSR in the 1^st^ year and then grew faster with higher SH and D0 values over the next 2 years. *P*. *yunnanensis* had the broadest variation in GR, SR in the 2^nd^ year, BSR in the 2^nd^ year, and D0 over 2 years among the three pines. Populations 1 and 2 rarely did bud set in October, and all plants in population 1 died during the 1^st^ winter. population 4 displayed the highest value of SRAW in the 2^nd^ year. Population 7 possessed higher RLC in red, BSR over 2 years, and SGR for 2 years than the other populations.

**Fig 3:**
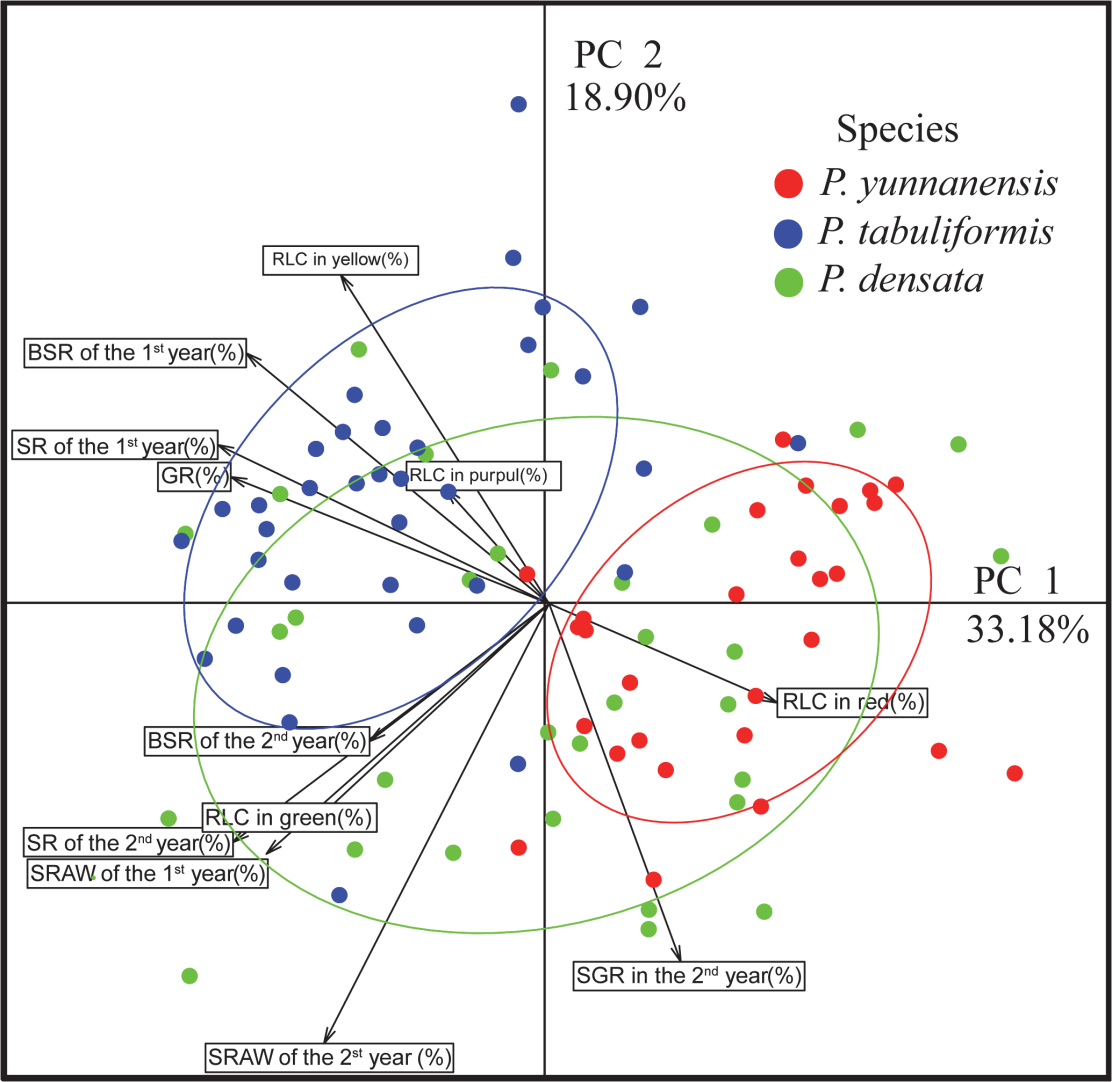
Scatter diagram of the first two principal coordinates (PC1 and PC2) from PCA for the three pine species based on adaptive indices and growth indices.

Principal components analysis (PCA) on seedling tested traits revealed four principal components with eigenvalues greater than 1. The four principal components accounted for 74.7% of the variation. Individually, the traits of GR, SR over 2 years, and BSR over 2 years, had high loadings in the 1st component, while RLC in green had high loadings in the 2nd component (Table 5). The first two components explained 52.1% of the variation (Fig. 3). Both *P*. *tabuliformis* and *P*. *yunnanensis* were clearly distinguished from each other. *P*. *densata* had a broad distribution that covered almost all of the area between the two parent species. Significant differences were observed between *P*. *tabuliformis* and *P*. *yunnanensis*. *P*. *densata* appeared to inherit adaptive characteristics of the parent species, suggesting that greater variation is present in the hybrid species

**Table 5:** Loadings on the first four components in PCA.

	Comp1	Comp2	Comp3	Comp4
GR...	−0.737	0.222	0.158	−0.190
SR of the 1^st^ year	−0.767	0.278	−0.220	−0.061
SRAW of the 1^st^ year	−0.655	−0.438	−0.439	0.190
SR of the 2^nd^ year	−0.732	−0.417	−0.359	0.191
SRAW of the 2st year	−0.521	−0.770	0.076	−0.036
BSR of the 1^st^ year	−0.702	0.438	0.193	−0.208
BSR of the 2^nd^ year	−0.416	−0.241	0.371	−0.489
SGR in the 2^nd^ year	0.305	−0.627	−0.083	0.201
RLC in red	0.529	−0.173	−0.344	−0.461
RLC in yellow	−0.483	0.575	−0.074	0.501
RLC in purpul	−0.239	0.201	−0.682	−0.457
RLC in green	−0.533	−0.374	0.496	−0.047
total	0.332	0.189	0.118	0.093

**Fig 4:**
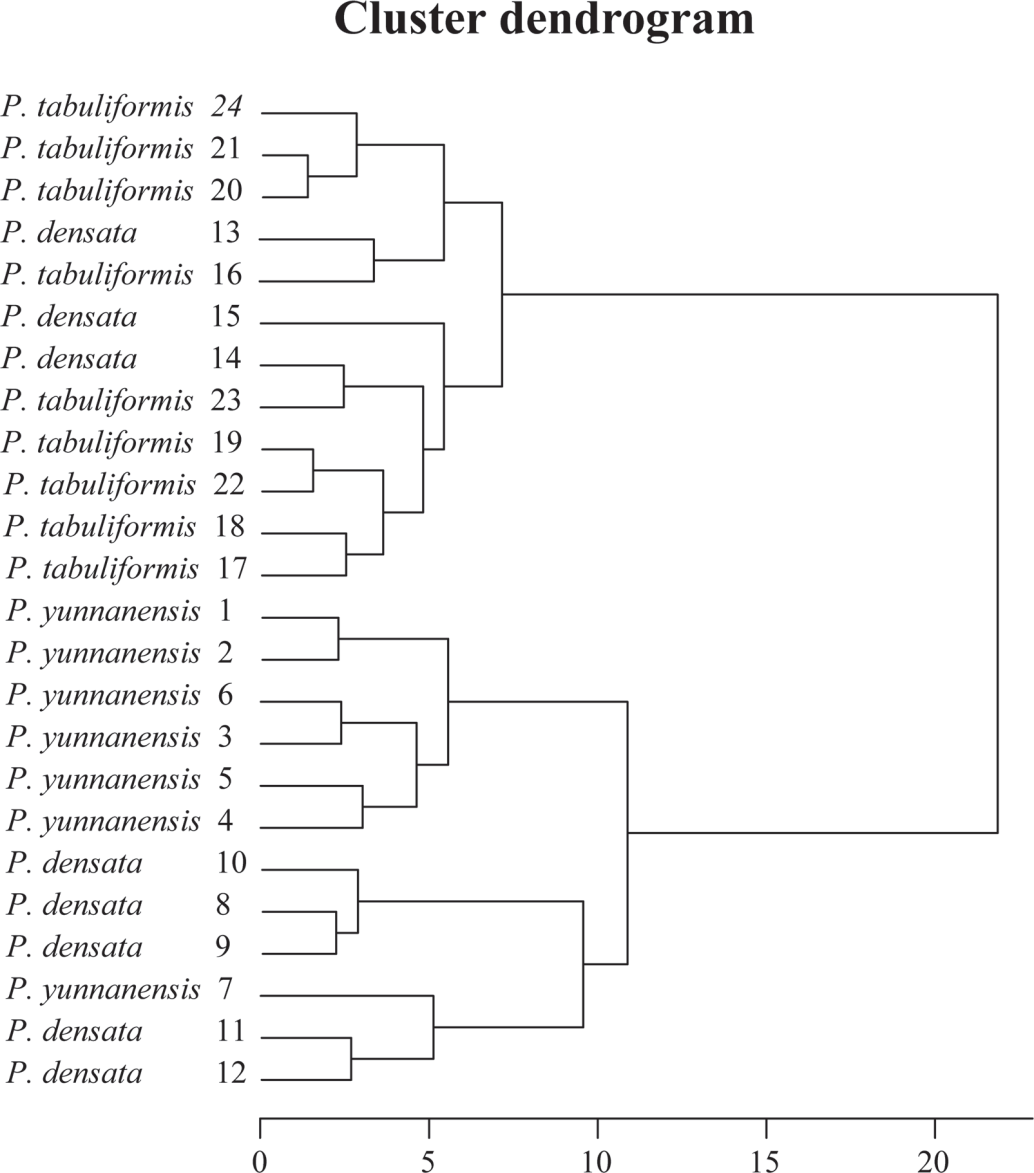
Result of the cluster analysis of the sampled populations for the three pine species based on adaptive indices and growth. Populations 1–7 are *P*. *yunnanensis*, populations 8–15 are *P*. *densata*, populations 16–24 are *P*. *tabuliformis*.

Based on eight weakly related characteristics (Pearson correlation coefficient lower than 0.5), hierarchical cluster analysis using the ward method produced two clearly defined groups within the 24 populations (Fig. 4). Populations of *P*. *tabuliformis* and *P*. *yunnanensis* were completely separated. Populations of *P*. *densata* were divided into four clusters: *P*. *densata* east 1 (14, 15), *P*. *densata* east 2 (13), *P*. *densata* central (11 12), and *P*. *densata* west (8, 9, 10). Populations of 13, 14, and 15 in *P*. *densata* were clustered in the *P*. *tabuliformis* group showing a closer relationship to *P*. *tabuliformis*, while populations 8, 9, 10, 11, and 12 in *P*. *densata* were clustered in the *P*. *yunnanensis* group and showed a closer relationship to *P*. *yunnanensis*. Population 7, located in the northeastern-most region of the *P*. *yunnanensis* range, had little differentiation from *P*. *densata*. A relationship may exist between population 7 and *P*. *densata*.

### Environmental association

For *P*. *yunnanensis*, annual average temperature and average temperature of the coldest month were associated with BSR over 2 years and RLC in red. Annual precipitation was significantly correlated with seedling survival (SR, SRAW), growth (D0 and SH), and needle traits (RLC) in the 1^st^ year. The average temperature of coldest month was positively correlated with the BSR over 2 years (Table 6).

**Table 6:** Correlation coefficient between measured traits and geographical variables of *P*. *yunnanensis*, *P*. *densata* and *P*. *tabuliformis*.

	*P*. *yunnanensis*				*P*. *densata*				*P*. *tabuliformis*		
Species	AT	AP	ATJ		AT	AP	ATJ		AT	AP	ATJ
GR	−0.1	−0.40*	−0.06		0.28	−0.18	0.21		−0.25	−0.39*	−0.14
SR of the 1st year	0.06	0.15	0.06		−0.16	0.12	−0.3		−0.2	−0.44**	−0.47**
SRAW of the 1st year	−0.15	−0.42*	−0.16		0.11	−0.27	0		0.16	0.07	0.06
SR of the 2nd year	−0.05	−0.32	−0.05		0.29	−0.35*	0.17		−0.1	−0.03	−0.05
SRAW of the 2nd year	−0.2	−0.27	−0.2		0.55**	−0.65**	0.50**		0.21	0.33	0.26
BSR of the 1st year	−0.44*	−0.31	−0.44*		−0.32	0.09	−0.42*		−.45**	−0.72**	−0.74**
BSR of the 2nd year	−0.67**	−0.49**	−0.69**		0.32	−0.39*	0.26		0.23	0	0.05
SGR in the 2nd year	−0.37*	−0.22	−0.39*		0.37*	−0.27	0.40*		0.42*	0.57**	0.58**
RLC in red	−0.56**	−0.47*	−0.56**		−0.39*	0.11	−0.22		0.08	0.29	0.14
RLC in yellow	0.02	0.15	0.04		−0.02	0.43*	−0.22		−0.03	−0.21	0.05
RLC in purple	−0.19	−0.22	−0.19		−0.3	0.18	−0.16		0.18	0.08	−0.1
RLC in green	0.63**	0.52**	0.63**		0.56**	−0.61**	0.51**		−0.12	0.09	−0.03
SH of the 1st year	0.03	0.21	0.02		0.52**	−0.40*	0.56**		0.13	0.05	0.16
SH of the 2nd year	0.07	0.26	0.07		0.45*	−0.39*	0.47*		0.1	0.09	0.22
D0 of the 1st year	0.54**	0.61**	0.53*		0.14	−0.46*	0.2		0.02	0.19	0.2
D0 of the 2nd year	0.02	0.37	0		0.49**	−0.12	0.46*		−0.14	−0.03	0.1

AT, Annual average Temperature; AP, Annual Precipitation; ATC, Average temperature of the coldest month.

* *P* < 0.05;

** *P* < 0.01.

For *P*. *densata*, annual average temperature was significantly positively correlated with growth rate of the 2^nd^ years, annual precipitation was significantly correlated with seedling survival (SR, SRAW), growth (D0 and SH), and needle traits (RLC) of the 2^nd^ years. Similarly, the average temperature of the coldest month was significantly positively correlated with seedling survival (SR, SRAW), phenology (BSR) and growth rate (D0 and SH) (Table 6).

For *P*. *tabuliformis*, the annual average temperature and the average temperature in January were significant correlation with phenology (BSR). Additionally, the average precipitation was significantly correlated to germination rate (GR) (Table 6).

## Discussion

### *P*. *densata* shows better fitness than parent species on the Tibetan Plateau

Speciation of *P*. *densata* was supposed to be the result of niche adaption [1,5,9]. Common garden experiment in hybrid habitat is one of the most direct approaches to investigation of juvenile fitness [29]. We found *P*. *densata* had the highest survival rate and the fastest growth rate compared to the two parental species which is indicative of an adaptive advantage over the parental species in the hybrid niche. Plant germination and seedling growth is environmentally demanding [30,31]. Traits for survival and development are the most direct indicators of seedling adaptation [32–34]. Although lower than *P*. *tabuliformis* in the GR, the survival rate (SR) of *P*. *densata* surpassed both parental species, Species with higher survival rate will have advantage in interspecific competitions [35,36]. Habitat factors on the Tibetan Plateau, such as precipitation, temperature, light, soil, and stress, significantly affected the fitness of seedling plants [9].

For traits associated with phenology, *P*. *densata* was intermediate between parent species. Bud set is a feature of woody plant for avoiding winter injury, which is induced by photoperiod and temperature [37–41]. The much earlier and higher ratio of bud set of *P*. *tabuliformis* is the results of adaptation of higher latitude condition in north China [42], while the much later and lower bud set rate of *P*. *yunnanensis* is the results of adaptation of lower latitude condition in south China [43]. The phenology of bud set in *P*. *densata* showed adaptation of a lower latitude and higher altitude condition of the Tibetan Plateau. The higher BSR of *P*. *densata* than *P*. *yunnannesis* will provide better cold resistance, and the later bud set than *P*. *tabuliformis* will allow for more shoot growth. *P*. *densata* also showed the highest rate of the secondary growth, which suggests that most plants in this species have the latent growth in years with favorable climate conditions. This growth strategy is a benefit for the species to gain a competitive advantage in the hybrid niche.

The changes of seedling color reflect the status of plants responding to lower temperature in autumn [13]. Yellowing of seedling needles is suggestive of plant death, while needles changing color from green to red or purple may be a protective mechanism of plants against environmental stresses from ultraviolet radiation and cold [44–50]. For these three pine species, *P*. *densata* had higher value of RCL in purple, and *P*. *yunnanensis* had higher value of RCL in red, suggesting that the two species could make an effective response to strong ultraviolet rays. A higher yellowing rate was found in *P*. *tabuliformis* seedlings, reflecting their maladaptation to high altitude habitat. All lines of evidence from this study provided support to a better fitness of *P*. *densata* than parental species on the Tibetan Plateau through utilizing a longer growth season, better survival, moderate bud set time and the protection from physiological mechanism in in hybrid habitat. The fitness advantage of *P*.*densata* likely has contributed to the hybrid speciation.

### Adaptation to different climate factors in the species complex

We included 16 representative populations of *P*. *tabuliformis* and *P*. *yunnanensis* in the field testing to provide a comprehensive assessment to the adaption of parental species in hybrid habitat. There were significant differences in precipitation, heat, light and other climate factors between the habitats of *P*. *densata* and the parental species. Low winter temperatures can cause chilling injuries for species which typically grows in the warmer and humid niches in the subtropical zone such as *P*. *yunnanensis* [43]. For *P*. *yunnanensis*, water depletion and hypothermy may have increased the mortality of its seedlings on the plateau. *P*. *tabuliformis* suffers selection pressure from a variety of ecological factors [40]. The growth time of *P*. *tabuliformis* should be determined by bud set phase and secondary growth rate [41]. We showed that bud set rate and secondary growth rate were significantly correlated with latitude (S1 Table) and the climate factors (Table 4), suggesting that the climate factors could influence the adaptability of *P*. *tabuliformis* by affecting its bud set process. Blue and violet light on the Tibetan Plateau have strong inhibitory effects on plant growth [49,51–54]. Purple rates of *P*. *tabuliformis* and *P*. *yunnanensis* seedlings were significantly correlated with altitude, indicating that the changes of seedling colors might affect the development of the parental species in the Tibetan Plateau.

A few populations of parent species (e.g. populations 16,17,18 of *P*. *tabuliformis* and populations 3,4,6,7 of *P*. *yunnanensis*) performed generally well in hybrid habitat, suggesting the fitness of each population may be related to its geographical distribution. The difference in fitness among population within parent species might come from (1) *local adaption in parental habitat*. Populations 16, 17, and 18, which are from the south distribution region, grew much faster than the other populations of *P*. *tabuliformis*. Population located in lower latitude of *P*. *tabuliformis* set bud at a later time than other populations, which maybe lengthen the growing period of the shoot on Tibetan Plateau. Similarly, *P*. *yunnanensis* is typically distributed in the subtropical zone, but the habitats of populations 3, 4, 6, and 7 were colder and drier than the other populations. Seedlings fitness of these populations was relatively high, which may be due to the cold tolerance of these populations. (2) *Interspecific gene flow with P*. *densata*. In our test, the GR, BSR, and SR in population 16 of *P*. *tabuliformis* were similar to the eastern population of *P*. *densata*, while the GR, BSR, and RCL in population 7 in *P*. *yunnanensis* were similar to central population of *P*. *densata*. Reproductive barrier between these species is weak [55]. Population 16 is located very close to the northern range of *P*. *densata*, bidirectional gene flow between the *P*. *tabuliformis* and *P*. *densata* may be possible through pollen exchange [10,56,57]. For *P*. *yunnanensis*, the genetic background of population 7 was similar to that of *P*. *densata*, which may have resulted from introgression and gene flow [10,23].

In general, adaptation to different climate factors may determine the adaptive differentiation at seedling stages of *P*. *tabuliformis* and *P*. *yunnanensis* on Tibetan Plateau. Both *P*. *tabuliformis* and *P*. *yunnanensis* occupy vast areas and different habitats from the hybrid. When transplanted out of the habitat, ecological factors could set strong selections on the fitness of each species. Due to the wide distribution of each species, differentiation in fitness traits within species is expected.

### Differentiation in fitness traits among *P*. *densata* populations

We included 8 populations of *P*. *densata* in the field trial. We found adaptive differences among these geographic populations which reflected an altitude gradient of selection. The results from clustering analysis showed that populations from the west, the middle and the east of *P*. *densata* could be divided into three distinct groups, suggesting that the populations in each geographical region had different evolutionary process, corroborating with the molecular investigations on the evolutionary history of *P*. *densata* on the plateau [12]. Moreover, the east groups adapted to the western habitat generally well even though it is more than 1000 km away from its geographic origin. However, populations 11 and 12, from the central range of *P*. *densata* adapted to the western habitat poorly. In the field testing, continuous variation in SH and D0 was found associated with altitude. Similar altitude effects on the evolution of other species have been reported for the eastern Tibetan Plateau [58–60]. We also noticed similar performance between adjacent populations of different species, which might be explained by pollen-mediated introgression [12; 16]. In our test the traits GR, BSR, and SR in eastern *P*. *densata* were similar to *P*. *tabuliformis*, while GR, BSR, and RCL in central *P*. *densata* were similar to *P*. *yunnanensis*. Molecular investigations show bidirectional gene flow between different species in the above regions [12,15,16]. Bidirectional gene flow between the hybrid species and either parent species may be occur via pollen flow when there is only weak crossability barrier among species [22,54,61]. The performance of the introgressed populations will be affected by location adaption as well as the gene flow.

## Conclusions

As one of the most documented examples of a homoploid hybrid speciation, *P*. *densata* evolved a unique adaptation in the high plateau environment. The findings from the present study support the hypothesis of previous authors that habitat divergence promoted the speciation of *P*. *densata* on the Tibetan Plateau. Although results of our experiments can only partially identify the adaptive features of the three pine species placed in the habitat of *P*. *densata*, our data supports the general findings from molecular studies. Homoploid hybrid speciation can be driven by chromosomal and recombinational variation and ecological selection. Field test on fitness related traits provides direct evidence complementing the theory of homoploid hybrid speciation. Our present study together with more long-term field experiment will be helpful for understanding of speciation process of *P*. *densata*.

## Supporting Information

S1 Table Correlation coefficient between measured traits and altitude for *P*. *yunnanensis*, *P*. *densata* and *P*. *tabuliformis*. (DOCX)

S2 Table Correlation coefficient between geographical variables and the climate factors. (DOCX)
