# Supplementary material for: Adaptive Differentiation in Seedling Traits in a Hybrid Pine Species Complex, Pinus densata and Its Parental Species, on the Tibetan Plateau
Source: PLoS One. 2015 Mar 10;10(3):e0118501. doi: 10.1371/journal.pone.0118501 (PMC4355066; doi:10.1371/journal.pone.0118501)
Supplement: S1 Table — (DOCX) [file pone.0118501.s001.docx]

**S1 Table. Correlation coefficient between measured traits and elevation for *P. yunnanensis*, *P. densata* and *P. tabuliformis*.**

| Species | *P. yunnanensis* | *P. densata* | *P. tabuliformis* |
| --- | --- | --- | --- |
| GR | 0.29 | -0.08 | 0.17 |
| SR of the 1st year | -0.1 | -0.39* | -0.2 |
| SRAW of the 1st year | 0.32 | -0.16 | -0.15 |
| SR of the 2nd year | 0.25 | -0.14 | -0.02 |
| SRAW of the 2nd year | 0.33 | 0.11 | -0.04 |
| BSR of the 1st year | 0.38* | -0.24 | -0.18 |
| BSR of the 2nd year | 0.59** | -0.02 | -0.16 |
| SGR in the 2nd year | 0.32 | 0.17 | 0.15 |
| RLC in red | 0.54** | 0.39* | 0.02 |
| RLC in yellow | -0.1 | -0.68** | 0.09 |
| RLC in purple | 0.27 | 0.34 | -0.42* |
| RLC in green | -0.64** | 0.08 | 0.18 |
| SH of the 1st year | -0.13 | 0.27 | -0.03 |
| SH of the 2nd year | -0.11 | 0.23 | 0.22 |
| D0 of the 1st year | -0.56** | 0.33 | 0.24 |
| D0 of the 2nd year | -0.17 | -0.09 | 0.25 |

* P < 0.05; ** P < 0.01.
