# Supplementary material for: Adaptive Differentiation in Seedling Traits in a Hybrid Pine Species Complex, Pinus densata and Its Parental Species, on the Tibetan Plateau
Source: PLoS One. 2015 Mar 10;10(3):e0118501. doi: 10.1371/journal.pone.0118501 (PMC4355066; doi:10.1371/journal.pone.0118501)
Supplement: S2 Table — (DOCX) [file pone.0118501.s002.docx]

**S2 Table. Correlation coefficient between geographical variables and the climate factors.**

|  | AT | AP | ATC |
| --- | --- | --- | --- |
| LON | -.620** | .628** | -.793** |
| LAT | -0.308 | 0.274 | -.543** |
| ELE | -0.157 | -.600** | 0.12 |

* P < 0.05; ** P < 0.01.

LON, Longitude; LAT, Latitude; ELE, Elevation; AT, Annual average Temperature; AP, Annual Precipitation; ATC, Average temperature of the coldest month
